# AMPA Receptor-Mediated Ca^2+^ Transients in Mouse Olfactory Ensheathing Cells

**DOI:** 10.3389/fncel.2019.00451

**Published:** 2019-10-04

**Authors:** Antonia Beiersdorfer, Christian Lohr

**Affiliations:** Division of Neurophysiology, University of Hamburg, Hamburg, Germany

**Keywords:** olfactory ensheathing cells, AMPA receptor, calcium, patch clamp, GluA2

## Abstract

Ca^2+^ signaling in glial cells is primarily triggered by metabotropic pathways and the subsequent Ca^2+^ release from internal Ca^2+^ stores. However, there is upcoming evidence that various ion channels might also initiate Ca^2+^ rises in glial cells by Ca^2+^ influx. We investigated AMPA receptor-mediated inward currents and Ca^2+^ transients in olfactory ensheathing cells (OECs), a specialized glial cell population in the olfactory bulb (OB), using whole-cell voltage-clamp recordings and confocal Ca^2+^ imaging. By immunohistochemistry we showed immunoreactivity to the AMPA receptor subunits GluA1, GluA2 and GluA4 in OECs, suggesting the presence of AMPA receptors in OECs. Kainate-induced inward currents were mediated exclusively by AMPA receptors, as they were sensitive to the specific AMPA receptor antagonist, GYKI53655. Moreover, kainate-induced inward currents were reduced by the selective Ca^2+^-permeable AMPA receptor inhibitor, NASPM, suggesting the presence of functional Ca^2+^-permeable AMPA receptors in OECs. Additionally, kainate application evoked Ca^2+^ transients in OECs which were abolished in the absence of extracellular Ca^2+^, indicating that Ca^2+^ influx via Ca^2+^-permeable AMPA receptors contribute to kainate-induced Ca^2+^ transients. However, kainate-induced Ca^2+^ transients were partly reduced upon Ca^2+^ store depletion, leading to the conclusion that Ca^2+^ influx via AMPA receptor channels is essential to trigger Ca^2+^ transients in OECs, whereas Ca^2+^ release from internal stores contributes in part to the kainate-evoked Ca^2+^ response. Endogenous glutamate release by OSN axons initiated Ca^2+^ transients in OECs, equally mediated by metabotropic receptors (glutamatergic and purinergic) and AMPA receptors, suggesting a prominent role for AMPA receptor mediated Ca^2+^ signaling in axon-OEC communication.

## Introduction

Ca^2+^ signaling in glial cells is involved in various intercellular processes such as the release of gliotransmitters, modulation of synaptic transmission, long-range Ca^2+^ wave propagation, and neurovascular coupling (Haydon, 2001; Carmignoto and Gomez-Gonzalo, 2010). Moreover, intracellular processes such as apoptosis, transcription as well as posttranslational modification are regulated by Ca^2+^ signaling (McConkey and Orrenius, 1997; Flavell and Greenberg, 2008). In glial cells, rises of intracellular Ca^2+^ are predominantly triggered by Ca^2+^ release from internal Ca^2+^ stores induced by metabotropic pathways (Deitmer et al., 1998; Verkhratsky et al., 1998), while Ca^2+^ influx was reported in rare cases in specialized astrocyte-like glial cells such as cerebellar Bergmann glia and retinal Müller glia (Burnashev et al., 1992; Muller et al., 1992; Wakakura and Yamamoto, 1994). However, more recent studies suggest that astroglial cells of different regions of the brain also express ligand-gated ion channels that may trigger Ca^2+^ responses via Ca^2+^ influx from the extracellular space (Schipke et al., 2001; Lalo et al., 2006; Mishra et al., 2016; Droste et al., 2017). In addition, store-operated channels as well as transient receptor potential (TRP) channels contribute to Ca^2+^ signaling in astrocytes (Singaravelu et al., 2006; Rungta et al., 2016; Belkacemi et al., 2017; Rakers et al., 2017; Toth et al., 2019). These studies indicate that Ca^2+^ influx might play a previously underestimated role in glial cell physiology and function. Olfactory ensheathing cells (OECs) represent a specialized population of glial cells, exclusively located in the olfactory nerve layer (ONL) in the olfactory bulb (OB) and the peripheral olfactory mucosa (Su and He, 2010; Lohr et al., 2014). They support growth and guidance of axons of olfactory sensory neurons (OSN) from the olfactory epithelium (OE) into the main OB (Graziadei and Graziadei, 1979; Doucette, 1984; Yang et al., 2015). It is assumed, that OECs generate an environment allowing the lifelong re-growth and re-integration of axons into the cellular network after degeneration by the expression of different neurite growth factors and cell adhesions molecules (Crandall et al., 2000; Woodhall et al., 2001; Cao et al., 2007; Yang et al., 2015). In addition, studies showed that Ca^2+^ signaling in OECs is a critical regulator for neurite outgrowth in OEC/retinal ganglion cell (RGC) co-cultures, suggesting a prominent role for Ca^2+^ signaling in OEC-axon interaction (Hayat et al., 2003). Extrasynaptic release of glutamate and ATP by OSN axons initiates Gq-mediated Ca^2+^ release from internal stores in OECs via mGluR1 and P2Y1 receptors (Rieger et al., 2007; Thyssen et al., 2010). Montague and Greer (1999) investigated the distribution of the ionotropic AMPA receptor subunits, GluA1, GluA2/3 and GluA4 in the ONL and found expression by nerve-associated glial cells. However, whether OECs exhibit functional AMPA receptors, which might conduct membrane currents and mediate Ca^2+^ influx is not known. We performed whole-cell voltage-clamp recordings, showing that kainate application induced AMPA receptor-mediated inward currents in OECs, which are partly mediated by Ca^2+^-permeable AMPA receptors. Kainate also induced Ca^2+^ transients that depended on Ca^2+^ influx via the receptor channel itself, but additionally comprised Ca^2+^ release from internal stores. Electrical stimulation of OSN axons evoked Ca^2+^ transients mediated by metabotropic receptors as well as Ca^2+^-permeable AMPA receptors, suggesting a role for AMPA receptor-mediated Ca^2+^ signaling in axon-OEC communication.

## Materials and Methods

### Animals and Olfactory Bulb Preparation

Mice of the GLAST-Cre^ERT2^ × tdTomato^fl/fl^, PLP-Cre^ERT2^ × tdTomato^fl/fl^ (age: p28–60) and NMRI (age: p18–p21; Naval Medical Research Institute) strains (Leone et al., 2003; Mori et al., 2006; Madisen et al., 2010) were kept at the institutional animal facility of the University of Hamburg. These mice expressed Cre recombinase under control of the promoters of the glutamate/aspartate transporter EAAT1 (GLAST; expressed by OECs and astrocytes) and the proteolipid protein (PLP; expressed by OECs and oligodendrocytes), respectively (Droste et al., 2017; Beiersdorfer et al., 2019). Animal rearing and all experimental procedures were performed according to the European Union’s and local animal welfare guidelines (GZ G21305/591-00.33; Behörde für Gesundheit und Verbraucherschutz, Hamburg, Germany). To induce reporter expression in GLAST-Cre^ERT2^ × tdTomato^fl/fl^ and PLP-Cre^ERT2^ × tdTomato^fl/fl^ mice, tamoxifen (Carbolution Chemicals GmbH, St. Ingbert, Germany) was dissolved in 8% ethanol/92% Mygliol^®^812 (Sigma Aldrich, Taufkirchen, Germany) and injected intraperitoneally for three consecutive days (starting p21; 100 mg/kg bodyweight). Animals were analyzed 7–12 days after the first injection. OBs were prepared as described previously (Stavermann et al., 2012). Both OBs were removed from the opened head in cool preparation solution (molarities in mM: 83 NaCl, 1 NaH_2_PO_4_x2H_2_O, 26.2 NaHCO_3_, 2.5 KCl, 70 sucrose, 20 D-(+)-glucose, and 2.5 MgSO_4_ × 7 H_2_O). Standard artificial cerebrospinal fluid (ACSF) for experiments and storage of the preparations consisted of (molarities in mM): 120 NaCl, 2.5 KCl, 1 NaH_2_PO_4_x2H_2_O, 26 NaHCO_3_, 2.8 D-(+)-glucose, 1 MgCl_2_, and 2 CaCl_2_). Preparation solution and ACSF were continuously perfused with carbogen (95% O_2_ and 5% CO_2_) to maintain the pH of 7.4 and to supply oxygen.

### Reagents

The compounds (2*S*,3*S*,4*S*)-3-(Carboxymethyl)-4-(prop- 1-en-2-yl)pyrrolidine-2-carboxylic acid (kainic acid, agonist of AMPA/kainate receptors), (*S*)-α-Amino-3-hydroxy-5- methylisoxazole-4-propionic acid, 4-(8-Methyl-9*H*-1,3-dioxolo [4,5-*h*][2,3]benzodiazepin-5-yl)-benzenamine hydrochloride (GYKI53655, antagonist of AMPA receptors), (E)-ethyl 1,1a,7,7a-tetrahydro-7-(hydroxyimino) cyclopropa[b]chromene-1a-carboxylate (CPCCOEt, antagonist of mGluR_1_), 2-methyl- 6-(phenylethynyl) pyridine (MPEP, antagonist of mGluR_5_), 2′-deoxy-N6-methyladenosine 3′,5′-bisphosphate (MRS2179, antagonist of P2Y_1_), 4-(2-[7-Amino-2-(2-furyl)[1,2,4]triazolo [2,3-α][1,3,5]triazin-5-ylamino]ethyl) pheno (ZM241385, antagonist of A_2__A_ receptors), (R)-(+)-7-chloro-8-hydroxy-3-methyl-1-phenyl-2,3,4,5-tetrahydro-1H-3-benzazepine (SCH2 3390, antagonist of D1-like dopamine receptors) and carbenoxolone disodium salt (CBX; inhibiting gap junctions) were obtained from Abcam (Cambridge, United Kingdom). The reagents D-2-amino-5-phosphonovaleric acid (D-APV; antagonist of NMDA receptors), N-[3-[[4-[(3-aminopropyl)amino]butyl] amino]propyl]-1-naphthaleneacetamide trihydrochloride (Naspm trihydrochloride antagonist of Ca^2+^-permeable AMPA receptors) and tetrodotoxin (TTX; inhibiting voltage-gated sodium channels) were received from Alomone labs (Jerusalem, Israel). All reagents were stored as stock solutions corresponding to the manufacturer’s instructions and added to ACSF directly before the experiment. Drugs were applied via the perfusion system driven by a peristaltic pump (Reglo MS4/12, Ismatec, Wertheim, Germany).

### Electrophysiology and Analysis

For electrophysiological experiments, OB slices (220 μm, horizontal) of GLAST-Cre^ERT2^ × tdTomato^fl/fl^ mice were prepared using a vibratome (Leica VT1200S, Nußloch, Germany). Slices were transferred into a recording chamber and continuously superfused with ACSF via the perfusion system. The experiments were performed at room temperature. Whole-cell voltage-clamp recordings (Multiclamp 700B, Molecular Devices) were performed on OECs, identified by tdTomato expression (excited at 543 nm, emission 553–618 nm), and the distinct localization in the ONL (Au et al., 2002). Recordings were digitized (Digidata 1440A, Molecular Devices) at 10–20 kHz and filtered (Bessel filter 2 kHz). The holding potential was −80 mV, achieved by current injection of −35.14 ± 13.5 pA on average. Patch pipettes had a resistance of 4–7 MΩ when filled with internal solution containing (mM): 105 K-gluconate, 30 KCl, 10 HEPES, 10 phosphocreatine, 4 Mg-ATP, 0.3 Na-GTP, 0.3 EGTA, and pH = 7.43. For visualization of the recorded OEC, ATTO-488 carboxy (20 μM, ATTO-Tech GmbH, Siegen, Germany) was added to the internal solution before the experiments. Agonists were applied via the perfusion system for 30 s. Antagonists were applied via the perfusion system 10 min prior to agonist application. The flow rate of the perfusion system amounted to approximately 2 mL/min. The experimental bath was oval in shape and had a diameter of 2.1 cm, the bath volume amounted to 1 mL. Therefore, agonist and antagonist concentrations continuously increased in the experimental bath, until the final concentration is reached. Vice versa, agonists were slowly washed out, accounting for long lasting effects. Kainate-induced inward currents in OECs were analyzed by measuring amplitude the currents.

### Ca^2+^ Imaging and Analysis

For Ca^2+^ imaging experiments, GLAST-Cre^ERT2^ × tdTomato^fl/fl^ and NMRI mice were used. Whole OBs were glued onto coverslips, transferred into a recording chamber and the coverslip secured with a U-shaped platinum wire. For multi-cell bolus loading (Stosiek et al., 2003), a glass pipette with a resistance of ∼3.5 MΩ (when filled with ACSF) was filled with 200 μM Fluo-8 AM (Thermo Fisher Scientific, Darmstadt, Germany) in ACSF, made from a 4 mM stock solution (dissolved in DMSO and 20% pluronic acid). After inserting the injection pipette into the ONL, the Ca^2+^ indicator was pressure-injected with 0.7 bar for 30 s into the tissue (PDES-01 AM, NPI electronic GmbH, Tamm, Germany), followed by an incubation of 20 min. Changes in the cytosolic Ca^2+^ concentration in OECs were detected by the fluorescence of Fluo-8 (excitation: 488 nm; emission: 500–530 nm) using a confocal microscope (eC1, Nikon, Düsseldorf, Germany). Images were acquired at a time rate of one frame every 3 s. A similar perfusion system and flow rate was used as described above. However, the experimental bath for Ca^2+^ imaging experiments was differently shaped (diameter 1 cm, volume 1.9 mL) resulting in differences in the kinetics of wash-in and wash-out of drugs between the electrophysiological and Ca^2+^ imaging experiments. To analyze changes in cytosolic Ca^2+^ in single cell somata, regions of interest (ROIs) were defined using Nikon EZ-C1 3.90 software. OECs were identified by GLAST promoter-driven tdTomato expression in GLAST-Cre^ERT2^ × tdTomato^fl/fl^ mice in most experiments. In wild type mice, OECs were identified by their distinct localization in the ONL. The changes in Ca^2+^ were recorded throughout the experiments as relative changes in Fluo-8 fluorescence (ΔF) with respect to the resting fluorescence, which was normalized to 100%. Quantification of the Ca^2+^ transients was achieved by calculating the amplitude of ΔF.

### Statistics

All values are stated as mean values ± standard error of the mean. The number of experiments is given as *n* = *x*, where *x* is the number of analyzed cells. At least 3 animals were analyzed for each set of experiments. Statistical significance was estimated by comparing three means using Friedmann ANOVA and the Wilcoxon *post hoc* test for paired data, and for comparing two means using the Mann–Whitney *U*-Test, with the error probability *p* (^∗^ *p* ≤ 0.05; ^∗∗^ *p* ≤ 0.01; ^∗∗∗^ *p* ≤ 0.001).

### Immunohistochemistry

Immunohistochemistry on OBs of PLP-Cre^ERT2^ × tdTomato^fl/fl^ mice (≥P28) was performed as described before (Droste et al., 2017). After dissection, the OBs were kept for 1 h at room temperature (RT) in formaldehyde (4% in PBS, pH 7.4). Afterward, 100 μm thick sagittal slices were prepared with a vibratome (VT1000S, Leica, Nußloch, Germany) and incubated for 1 h in blocking solution (10% normal goat serum (NGS), 0.5% Triton X-100 in PBS) at RT. Subsequently, the slices were incubated for 48 h at 4°C with the following primary antibodies: Guinea pig anti-GluA1 (Alomone labs; 1:200); rabbit anti-GluA2 (Millipore, 1:200); rabbit anti-GluA4 (Millipore; 1:200). To validate the specificity of the GluA antibodies we used cerebellar slices as control, since the distribution of GluA subunits is well documented in this brain area (Supplementary Figure S1). Moreover, the antibodies against GluA1 and GluA4 have been validated in glia-specific GluA1 and GluA4 double knockout mice before (Saab et al., 2012). In our control experiments, the GluA2 antibody only labeled cells known to express GluA2, but not cells that lack GluA2 such as Bergmann glial cells (Burnashev et al., 1992; Muller et al., 1992; Saab et al., 2012), as shown before for the used antibody (Droste et al., 2017). Hence, we consider the used antibodies as efficient and specific. The antibodies were diluted in 1% NGS, 0.05% TritonX-100 in PBS. Slices were incubated in PBS with the following secondary antibodies for 24 h at 4°C: goat anti-rabbit Alexa Fluor 488 (Thermo Fisher Scientific; 1:1000) or goat anti-guinea pig Alexa Fluor 488 (Thermo Fisher Scientific; 1:1000). Moreover, Hoechst 33342 (5 μM; Molecular Probes, Eugene, OR, United States) was added to stain nuclei. Slices were mounted on slides using self-hardening embedding medium (Immu-Mount, Thermo Fisher Scientific). Immunohistological stainings were analyzed using a confocal microscope (Nikon eC1). Confocal images were adjusted for brightness and contrast using ImageJ and Adobe Photoshop CS6.

## Results

### Distribution of GluA Subunits in the ONL

**FIGURE 1:**
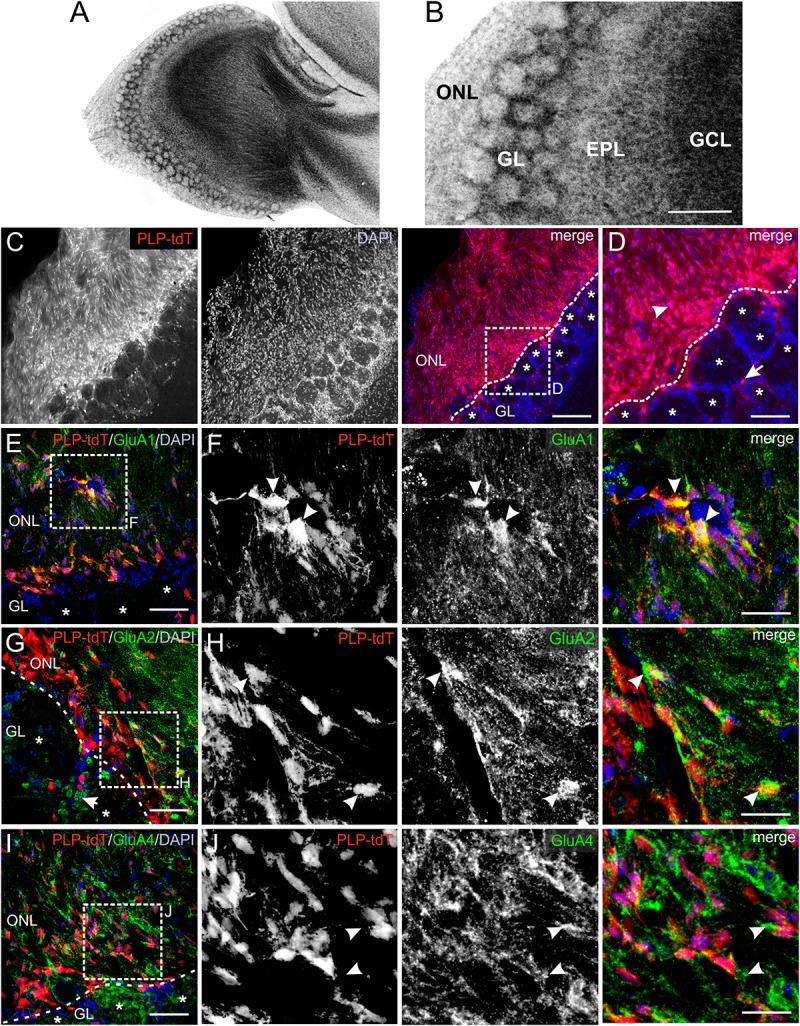
Distribution of AMPA receptor subunits in the olfactory nerve layer (ONL). **(A)** Laminar organization of the olfactory bulb. Nuclei were stained with Hoechst (5 μM). **(B)** The ONL, glomerular layer (GL), external plexiform layer (EPL) and granule cell layer (GCL) are clearly distinguishable. Scale bar: 200 μm. **(C)** Distribution of PLP-Cre-dependent tdTomato expression in the ONL and GL. Olfactory ensheathing cells (OECs) are located in the ONL, whereas tdTomato-expressing oligodendrocytes are located in the GL. Nuclei are stained with Dapi (5 μM; blue). The assumed border between the ONL and GL is indicated by the dotted line. Glomeruli are marked by asterisks. Scale bar: 150 μm. **(D)** Magnified view from (C). OECs are located in the ONL (arrowhead) and oligodendrocytes (arrow) in the GL visualized by PLP-Cre-dependent tdTomato expression (red). Scale bar: 50 μm. **(E)** Distribution of GluA1 immunoreactivity (green) in the ONL of PLP-Cre^ERT2^ × tdTomato^fl/fl^ mice (red). Nuclei are stained with Dapi (blue). Glomeruli are marked by asterisks. Scale bar: 50 μm. **(F)** Magnified view from (E). GluA1 immunostaining (green) colocalize with tdTomato-expressing OECs (red) in the ONL (arrowheads). Scale bar: 25 μm. **(G)** Distribution of GluA2 immunoreactivity (green) in the ONL of PLP-Cre^ERT2^ × tdTomato^fl/fl^ mice (red). GluA2 is widely distributed in the ONL, as well as in the GL. Nuclei are stained with Dapi (blue). Glomeruli are marked by asterisks. Scale bar: 50 μm. **(H)** Magnified view from (G). GluA2 immunoreactivity colocalizes with tdTomato-expressing OECs (red) in the ONL (arrowheads). Scale bar: 25 μm. **(I)** Distribution of GluA4 immunoreactivity (green) in the ONL of PLP-Cre^ERT2^ × tdTomato^fl/fl^ mice (red). Nuclei are stained with Dapi (blue). Glomeruli are marked by asterisks. Scale bar: 50 μm. **(J)** Magnified view from (I). GluA4 immunoreactivity (green) is sparsely colocalized with tdTomato-positive OEC somata (red), but is localized adjacent to OEC somata (arrowheads). Scale bar: 25 μm.

AMPA receptors constitute of four subunits (GluA1-GluA4), which form complex heteromeric cation channels, activated by glutamate or selective receptor agonists such as AMPA and kainate (Steinhauser and Gallo, 1996). The composition of the subunits is especially critical to determine the Ca^2+^ permeability of the channel. Thus, GluA2-lacking AMPA receptors show a high permeability for Ca^2+^ whereas GluA2-containing AMPA receptors are impermeable for Ca^2+^ (Hollmann et al., 1991). The OB is divided into several clearly distinguishable layers (Figures 1A,B) and immunoreactivity against GluA1, GluA2/3, and GluA4 demonstrated a wide distribution of these subunits in all layers, while mRNA expression analyses showed low amounts of GluA3 in the OB (Montague and Greer, 1999; Horning et al., 2004). We aimed to analyze the distribution of GluA1, GluA2, and GluA4 specifically in the ONL by immunohistochemistry using PLP-Cre^ERT2^ × tdTomato^fl/fl^ mice. The *plp* gene encodes for the PLP, expressed by oligodendrocytes, and its slice variant DM20, expressed by oligodendrocytes and OECs, enabling the specific identification of oligodendrocytes and OECs by Cre-recombinase-driven tdTomato expression (Griffiths et al., 1995; Dickinson et al., 1997). OECs are present exclusively in the ONL, whereas oligodendrocytes are located in the glomerular layer (GL) and deeper layers but not in the ONL (Figures 1C,D; Doucette, 1990, 1991). Therefore, PLP-Cre^ERT2^-driven tdTomato expression in the ONL is assumed to be restricted to OECs (Figures 1C,D; Beiersdorfer et al., 2019; Piantanida et al., 2019). GluA1 immunoreactivity was widely distributed in the entire ONL, colocalizing intensely with tdTomato-expressing OECs (Figures 1E,F). Homogenous GluA2 immunoreactivity was detected in the ONL, with scattered colocalization with tdTomato-positive OECs (Figures 1G,H). In the GL, GluA2-positive juxtaglomerular cells were present (Figure 1G, arrow; Droste et al., 2017). Additionally, GluA4 immunoreactivity was found in the ONL, however, it sparsely colocalized with tdTomato-positive OEC somata (Figures 1I,J). At higher magnification, GluA4 immunoreactivity was found in close approximation of tdTomato-expressing OECs, likely indicating GluA4 located in cell processes of OECs (Figure 1J, see arrowheads). The results suggest that OECs express GluA1 and GluA2, while GluA4 appears not to be present in OEC somata but presumably in cell processes.

### Kainate Induces AMPA Receptor-Mediated Inward Currents in OECs

**FIGURE 2:**
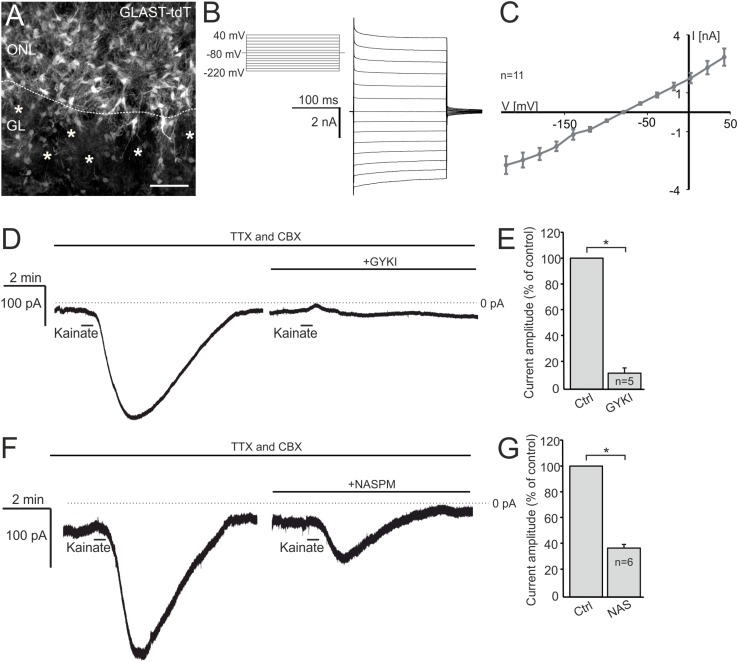
Kainate induces AMPA receptor-mediated inward currents in olfactory ensheathing cells (OECs). **(A)** Distribution of OECs in the olfactory nerve layer (ONL), identified by GLAST-Cre^ERT2^-driven tdTomato expression. Scale bar: 50 μm. **(B)** Voltage-step protocol applied to an OEC, recorded in whole cell voltage clamp mode. **(C)** All recorded OECs showed a linear current-voltage relationship. **(D)** The application of kainate (100 μM) induced an inward current in OECs, that was reduced by the selective AMPA receptor inhibitor GYKI 53655 (100 μM). All experiments were performed in the presence of TTX (1 μM) and CBX (150 μM). **(E)** Averaged amplitudes and statistical analysis of kainate-induced inward currents in OECs. **(F)** NASPM (100 μM), a selective inhibitor of Ca^2+^-permeable AMPA receptors, reduced kainate-induced inward currents significantly. **(G)** Averaged amplitudes and statistical analysis of kainate-induced inward currents in OECs. ^∗^*p* ≤ 0.05.

Functional AMPA/kainate receptors have been investigated in macroglial cells, namely astrocytes and oligodendrocyte precursor cells in gray and white matter as well as microglial cells (Burnashev et al., 1992; Muller et al., 1992; Jabs et al., 1994; Seifert and Steinhauser, 1995; Noda et al., 2000; Zonouzi et al., 2011; Hoft et al., 2014; Droste et al., 2017). Based on our immunohistochemical data, we suggested that OECs in the ONL might also express functional AMPA receptors. Therefore, we performed electrophysiological recordings in OECs in acute OB slices of GLAST-Cre^ERT2^ × tdTomato^fl/fl^ mice. OECs were identified by GLAST promoter-driven tdTomato expression in the ONL (Figure 2A). Additionally, a voltage-step protocol was applied to characterize the I/V relationship of the recorded cell. All OECs analyzed showed a characteristic linear I/V relationship (Figures 2B,C; Rela et al., 2010; Beiersdorfer et al., 2019). We used kainate to agonize AMPA/kainate receptors, since kainate induced a non-desensitizing AMPA receptor-mediated inward current in acutely isolated hippocampal glial cells (Seifert and Steinhauser, 1995). Kainate application (100 μM, 30 s) induced a prominent slowly rising and long lasting inward current with a mean amplitude of 231.5 ± 58.9 pA (at −80 mV holding potential) in OECs (Figure 2D). To specifically isolate kainate-induced responses in OECs, all experiments were performed in the presence of the gap junction inhibitor carbenoxolone (CBX, 100 μM) to avoid intercellular panglial communication to juxtaglomerular astrocytes that respond to kainate and might interfere with recordings in OECs (Droste et al., 2017; Beiersdorfer et al., 2019). Additionally, neuronal activity was suppressed by inhibiting voltage-gated sodium channels by tetrodotoxin (TTX, 1 μM). Application of GYKI53655 (100 μM), a selective AMPA receptor antagonist, significantly reduced kainate-induced inward currents in OECs by 88.8 ± 3.2% of the control (*n* = 5; *p* = 0.022), suggesting that the kainate-induced response is mainly mediated by AMPA receptors in OECs (Figures 2D,E). To further characterize the AMPA receptor subtype involved in kainate-induced inward currents, GluA2-lacking Ca^2+^-permeable AMPA receptors were inhibited by the selective antagonist, NASPM (100 μM) (Koike et al., 1997). NASPM reduced kainate-mediated inward currents in OECs significantly by 63.9 ± 2.7% of the control (*n* = 6; *p* = 0.019), suggesting that OECs exhibit functional Ca^2+^-permeable AMPA receptors (Figures 2F,G).

### Kainate-Mediated Ca^2+^ Transients in OECs Depend on Extracellular Ca^2+^

**FIGURE 3:**
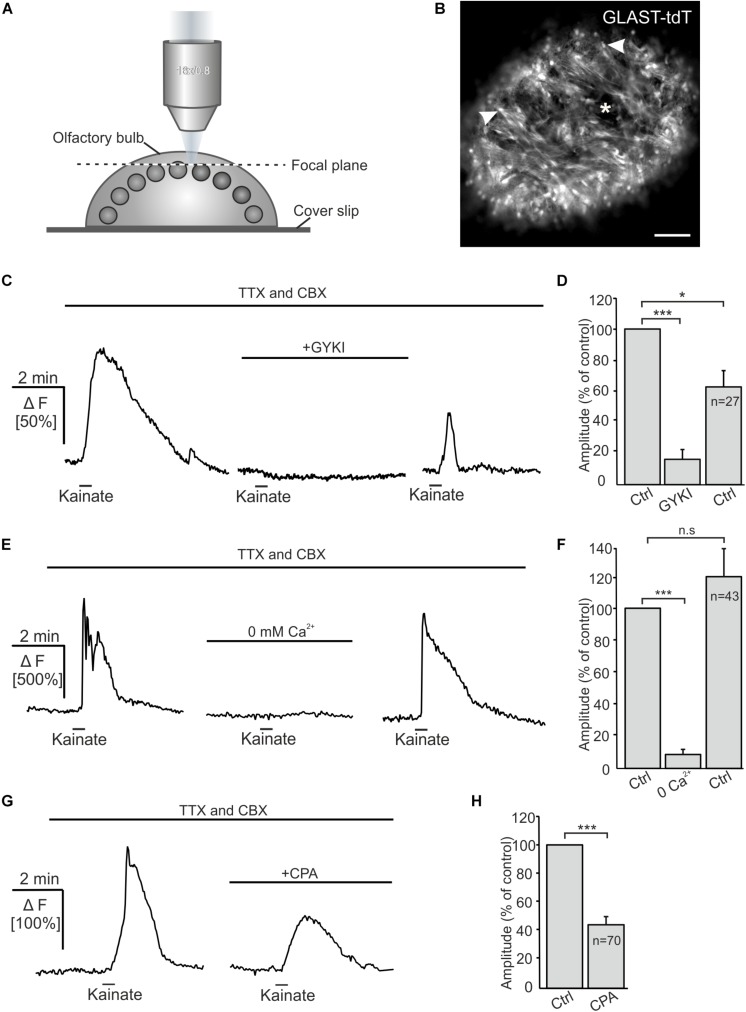
Kainate-induced Ca^2+^ transients in olfactory ensheathing cells (OECs) depend on extracellular Ca^2+^ and intracellular Ca^2+^ stores. **(A)** Schematic illustration of the experimental set up of Ca^2+^ imaging experiments. Olfactory bulb (OB) *in-toto* preparations of GLAST-Cre^ERT2^ × tdTomato^fl/fl^ mice were fixed and placed under the confocal microscope. Fluo-8 was pressure injected into the tissue. The focal plane as seen in panel **(B)** is indicated by the dotted line. **(B)** Confocal image of tdTomato-expressing OECs in an OB *in-toto* preparation. Arrowheads highlight OEC somata located in the ONL. One glomerulus is indicated by an asterisk. Scale bar: 100 μm. **(C)** Kainate-induced Ca^2+^ transients in OECs in the presence of TTX (1 μM) and CBX (150 μM) were reduced by GYKI 53655 (100 μM). **(D)** GYKI 53655 significantly reduced kainate-mediated Ca^2+^ transients. **(E)** Withdrawal of extracellular Ca^2+^ abolished kainate-induced Ca^2+^ transients in OECs completely. Restitution of extracellular Ca^2+^ caused a recovery of kainate-induced Ca^2+^ transients in OECs. **(F)** Averaged amplitudes and statistical analysis of kainate-mediated Ca^2+^ transients. **(G)** Upon intracellular Ca^2+^ store depletion by CPA (20 μM) kainate-induced Ca^2+^ transients were significantly reduced. **(H)** Averaged amplitudes and statistical analysis of the effect of CPA on kainate-mediated Ca^2+^ transients. ^∗∗∗^*p* ≤ 0.001; ^∗^*p* ≤ 0.05. n.s.: not significant.

Kainate-induced Ca^2+^ transients in Bergmann glial cells of the cerebellum result from Ca^2+^ influx directly through the AMPA receptor channel (Burnashev et al., 1992; Muller et al., 1992). However, AMPA/kainate receptors may also activate metabotropic pathways (Wang et al., 1997; Rozas et al., 2003; Takago et al., 2005). We performed confocal Ca^2+^ imaging experiments to investigate the source of Ca^2+^ that fuels Ca^2+^ transients in OECs in response to kainate application. The Ca^2+^ indicator Fluo-8 AM was pressure injected into OB *in-toto* preparations of GLAST-Cre^ERT2^ × tdTomato^fl/fl^ mice (Figures 3A,B). Experiments were performed in the presence of CBX and TTX to suppress indirect effects. Kainate application induced Ca^2+^ transients in OECs with a mean amplitude of 107.8 ± 5.3% ΔF. GYKI 53655 (100 μM), an AMPA receptor antagonist, greatly reduced kainate-evoked Ca^2+^ transients in OECs by 84.0 ± 6.1% of the control (*n* = 27; *p* = 5.93 × 10^–6^), showing that kainate-evoked Ca^2+^ transients are mediated by AMPA receptors (Figures 3C,D). Removal of extracellular Ca^2+^ reduced kainate-induced Ca^2+^ transients by 91.9 ± 2.3% (*n* = 43; *p* = 1.16 × 10^–8^). Kainate-evoked Ca^2+^ responses recovered after readdition of extracellular Ca^2+^, the average amplitude reached 122.0 ± 18.3% of the control (*p* = 0.16) (Figures 3E,F). To estimate the impact of intracellular Ca^2+^ stores on kainate-induced Ca^2+^ transients in OECs, we applied cyclopiazonic acid (CPA, 20 μM) to inhibit Ca^2+^ uptake into Ca^2+^ stores via SERCA (sarcoplasmic/endoplasmic reticulum calcium ATPase). Kainate-induced Ca^2+^ transients were significantly reduced by 57.6 ± 4.2% upon Ca^2+^ store depletion (*n* = 70; *p* = 8.18 × 10^–8^) (Figures 3G,H). Our results suggest that Ca^2+^ influx via AMPA receptor channels is essential to trigger Ca^2+^ transients in OECs, whereas Ca^2+^ release from internal stores is a secondary effect as a result of the kainate-evoked Ca^2+^ influx. To verify that the AMPA-evoked membrane current and associated Ca^2+^ influx was not elicited by a preceding Ca^2+^ increase, we evoked a Ca^2+^ transient by application of ADP and recorded membrane currents. ADP induced a large Ca^2+^ increase but failed to elicit membrane currents, demonstrating that Ca^2+^-dependent membrane conductances are negligible in OECs (Supplementary Figure S2).

### Endogenous Glutamate Release Induces AMPA Receptor-Mediated Ca^2+^ Transients in OECs

**FIGURE 4:**
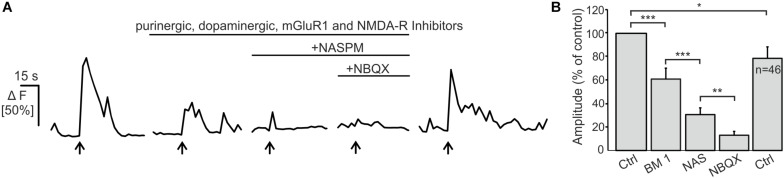
Electrical stimulation of OSN axons evokes AMPA-mediated Ca^2+^ transients in olfactory ensheathing cells (OECs).**(A)** Electrical stimulation of OSN axons evoked Ca^2+^ transients in OECs in control conditions (ACSF), and in the presence of purinergic (MRS2179, 100 μM; ZM241385; 0.5 μM), dopaminergic (SCH23390; 5 μM) an glutamatergic (CPCCOEt, 100 μM; D-APV, 100 μM) receptor antagonists. The additional application of NASPM (100 μM) further reduced Ca^2+^ transients in OECs. NBQX (20 μM) abolished stimulation-induced Ca^2+^ transients. **(B)** Averaged amplitudes of stimulation-evoked Ca^2+^ transients in OECs. ^∗∗∗^*p* < 0.0001; ^∗∗^*p* < 0.01; ^∗^*p* < 0.05.

Electrical stimulation of OSN axons evokes ectopic release of glutamate and ATP, which initiates mGluR1 and P2Y1 receptor-mediated Ca^2+^ rises in OECs (Berkowicz et al., 1994; Rieger et al., 2007; Thyssen et al., 2010). Here, we showed that OECs express functional Ca^2+^-permeable AMPA receptors which may also contribute to Ca^2+^ transients in response to neuronal activity. We endogenously released glutamate upon electrical stimulation of OSN axons (20 Hz, 2 s) and recorded Ca^2+^ transients in Fluo-8-loaded OECs in wild type animals. Electrical stimulation of OSN axons induced Ca^2+^ transients in OECs with an amplitude of 51.8 ± 5.0% ΔF (*n* = 46). By eliminating purinergic (MRS2179), dopaminergic (SCH23390) and mGluR1-mediated Ca^2+^ transients (CPCCOEt) as well as possible indirect NMDA-mediated neuronal responses (D-APV), the mean amplitude of stimulation-induced Ca^2+^ transients in OECs were significantly reduced to 61.0 ± 9.3% of the control (*p* = 3.68 × 10^–4^). Additional inhibition of Ca^2+^-permeable AMPA receptors by the selective antagonist NASPM further reduced the mean amplitude to 30.6 ± 5.8 (*p* = 1.36 × 10^–5^) of the control. NBQX almost completely abolished stimulation-induced Ca^2+^ transients in OECs and the amplitude further decreased significantly to 13.01 ± 3.3% of the control (*p* = 0.002). Receptor inhibition was reversible and stimulation-induced Ca^2+^ transients in OECs recovered after wash out (Figure 4). The results indicate that endogenous glutamate release triggers AMPA receptor-mediated Ca^2+^ responses in OECs, suggesting a possible role for AMPA receptors in axon-OEC communication.

## Discussion

In the present study, we investigated the role of AMPA receptors in OEC physiology and axon-OEC communication. Kainate induced inward currents and Ca^2+^ transients in OECs that were partially mediated by the GluA2-lacking Ca^2+^-permeable AMPA receptor subtype. Endogenous glutamate release initiated AMPA receptor-mediated Ca^2+^ transients in OECs, indicating the relevance of AMPA receptors for axon-OEC communication.

We showed clear GluA1 and GluA2 immunoreactivity in the ONL colocalized with tdTomato-expressing OECs, while GluA4 immunoreactivity rarely colocalized with tdTomato in OECs. However, in the present study tdTomato was predominantly localized in OEC somata, limiting the detection of OEC processes, in which GluA4 might be enriched, as it has been demonstrated in a study by Montague and Greer (1999), showing strong GluA4 immunoreactivity in cell processes of presumed olfactory nerve-associated glial cells. Therefore, the results of our and other studies suggest the presence of at least three of four GluA subunits in OECs.

While the presence of GluA2 in OECs argues against Ca^2+^ permeability of the AMPA receptors, our physiological data implies the involvement of Ca^2+^ influx via Ca^2+^-permeable AMPA receptors. AMPA receptor-dependent Ca^2+^ influx could either be conducted by Ca^2+^-permeable AMPA receptors or by voltage-gated Ca^2+^ channels activated by AMPA receptor-mediated depolarization (Porter and McCarthy, 1995). However, OECs do not generate voltage-dependent Ca^2+^ influx, e.g., via voltage-gated Ca^2+^ channels, ruling out that a depolarization by AMPA receptors is causative for the kainate-evoked Ca^2+^ signals (Thyssen et al., 2010). In addition, both kainate-evoked membrane currents and Ca^2+^ transients were largely reduced by NASPM, a selective blocker of Ca^2+^-permeable AMPA receptors (Koike et al., 1997). Hence, taking into account that on the one hand side OECs show immunoreactivity to the GluA2 subunit and on the other hand side the current and Ca^2+^ responses are NASPM-sensitive, the results suggest that OECs express both Ca^2+^-permeable as well as Ca^2+^-impermeable AMPA receptors. This is confirmed by the NASPM-insensitive but GYKI-sensitive fraction of kainate-evoked inward currents. In addition to Ca^2+^ influx via Ca^2+^-permeable AMPA receptors, Ca^2+^ release from internal stores contributes to AMPA receptor-mediated Ca^2+^ transients. OECs generate prominent Ca^2+^-induced Ca^2+^ release (CICR) and even a small Ca^2+^ increase generates a fast and large Ca^2+^ transient by CICR (Stavermann et al., 2012). Thus, Ca^2+^ influx through AMPA receptors upon kainate application could induce CICR that boosts the Ca^2+^ increase and accounts for the fast rising kinetics of the Ca^2+^ signal as compared to the rising phase of the kainate-evoked membrane current. We conclude that Ca^2+^ influx is the initial Ca^2+^ signal that triggers subsequent internal Ca^2+^ release such as CICR, as shown by the entire suppression of the Ca^2+^ signal by external Ca^2+^ withdrawal but only partial reduction upon Ca^2+^ store depletion.

Ca^2+^ signaling in OECs may serve different functions. In other glial cells such as astrocytes, increases in the cytosolic Ca^2+^ concentration has been reported to evoke release of so-called gliotransmitters that affect nearby neurons and synapses, but also release of vasoactive substances such as prostaglandins and arachidonic acid (Bazargani and Attwell, 2016; Guerra-Gomes et al., 2017). OECs enwrap blood vessels in the ONL (Herrera et al., 2005) and respond to neurotransmitters extrasynaptically released from axons of OSN with Ca^2+^ signals (Rieger et al., 2007; Thyssen et al., 2010; this study). Increases in intracellular Ca^2+^ in OECs results in constriction of adjacent capillaries (Thyssen et al., 2010). Hence, Ca^2+^ signals evoked by glutamate release from axons in the ONL and subsequent activation of Ca^2+^-permeable AMPA receptors may lead to vasoresponses that adjust blood flow to the metabolic demand upon action potential firing, a mechanism termed neurovascular coupling.

In summary, our results show that release of glutamate from axons of OSN results in Ca^2+^ transients in OECs that are partially mediated by AMPA receptors. AMPA receptor-mediated Ca^2+^ transients were due to Ca^2+^ influx, most likely through Ca^2+^-permeable AMPA receptors themselves, and subsequent Ca^2+^ release from internal Ca^2+^ stores.

## Data Availability Statement

The datasets generated for this study are available on request to the corresponding authors.

## Ethics Statement

The animal study was reviewed and approved by GZ G21305/591-00.33; Behörde für Gesundheit und Verbraucherschutz, Hamburg, Germany.

## Author Contributions

AB and CL designed the experiments and wrote the manuscript. AB performed the experiments and analyzed the data.

## Conflict of Interest

The authors declare that the research was conducted in the absence of any commercial or financial relationships that could be construed as a potential conflict of interest.

## Supplementary Material

FIGURE S1 Control stainings of the cerebellum to validate the specificity of anti-GluA antibodies. GluA1 was detected in Purkinje neurons (arrowhead) and Bergmann glial cells (arrow), GluA2 in Purkinje neurons and GluA4 in Bergmann glial cells, in line with published data. Scale bars: 50 μm.

FIGURE S2 ADP evokes Ca^2+^ transients but not membrane currents. **(A)** The application of kainate (100 μM) induced an inward current in OECs, whereas the application of ADP (100 μM) did not. **(B)** Average current amplitudes of kainate and ADP-induced currents in OECs. **(C)** Application of kainate and ADP induced Ca^2+^ transients in OECs. **(D)** Average amplitudes of kainate- and ADP-evoked Ca^2+^ transients.
