# Supplementary figures and images for: AMPA Receptor-Mediated Ca2+ Transients in Mouse Olfactory Ensheathing Cells
Source: Front Cell Neurosci. 2019 Oct 4;13:451. doi: 10.3389/fncel.2019.00451 (PMC6788192; doi:10.3389/fncel.2019.00451)

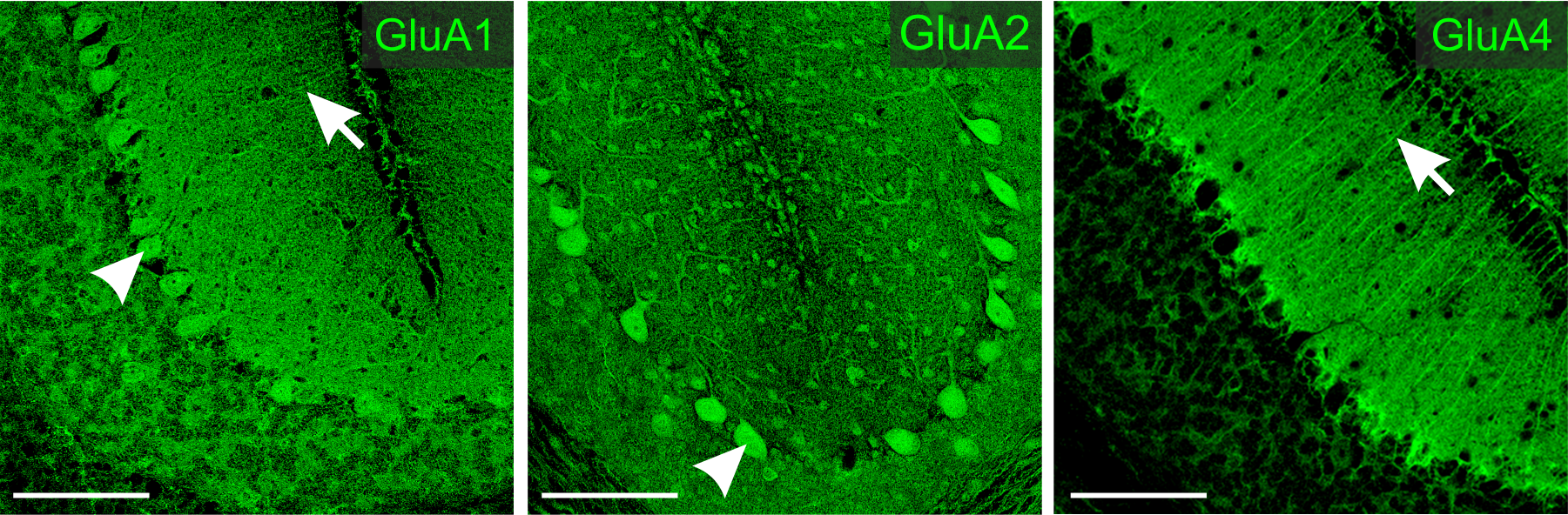

Supplement: FIGURE S1 — Control stainings of the cerebellum to validate the specificity of anti-GluA antibodies. GluA1 was detected in Purkinje neurons (arrowhead) and Bergmann glial cells (arrow), GluA2 in Purkinje neurons and GluA4 in Bergmann glial cells, in line with published data. Scale bars: 50 μm. [file Image_1.TIF]

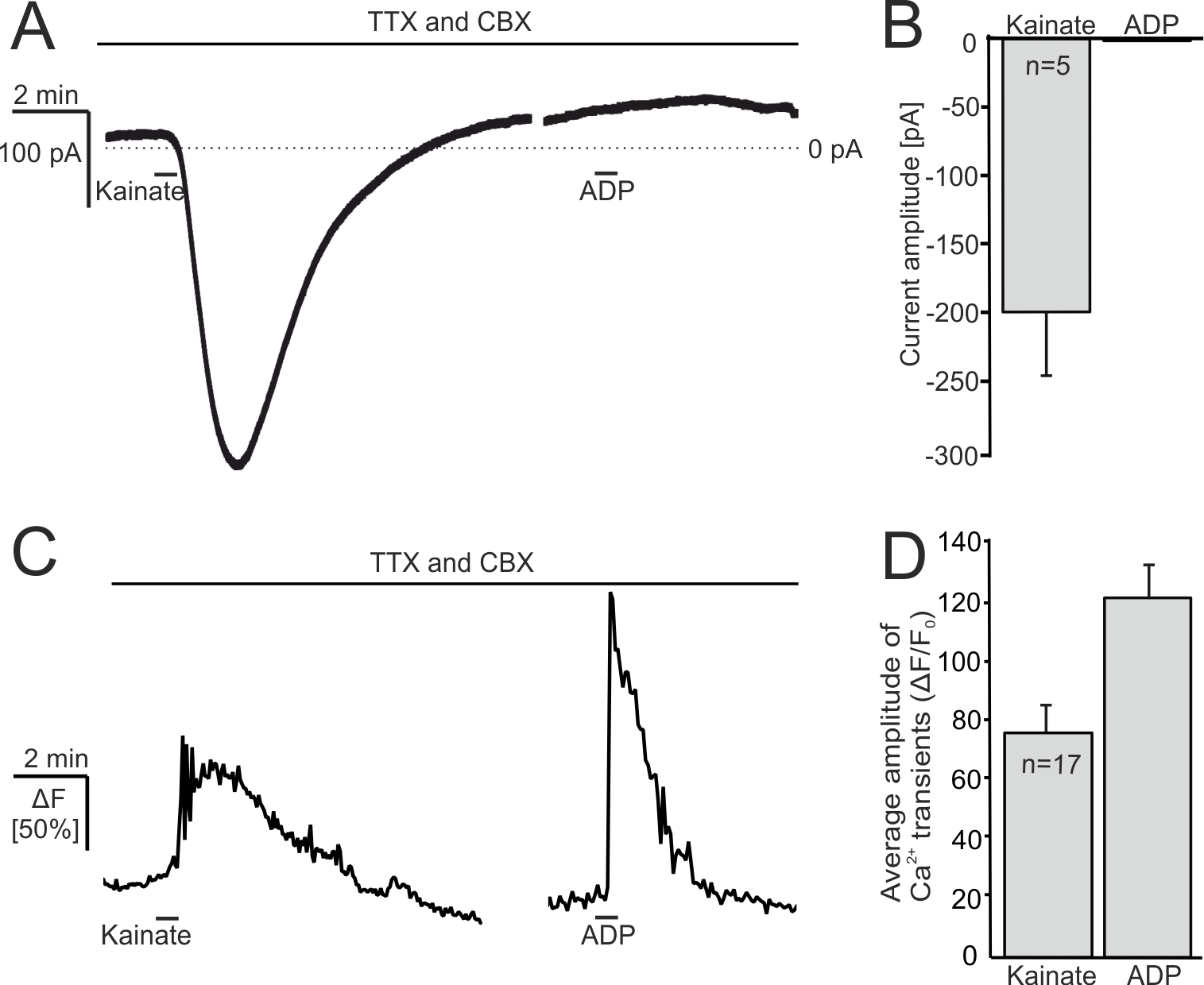

Supplement: FIGURE S2 — ADP evokes Ca2+ transients but not membrane currents. (A) The application of kainate (100 μM) induced an inward current in OECs, whereas the application of ADP (100 μM) did not. (B) Average current amplitudes of kainate and ADP-induced currents in OECs. (C) Application of kainate and ADP induced Ca2+ transients in OECs. (D) Average amplitudes of kainate- and ADP-evoked Ca2+ transients. [file Image_2.TIF]
